# In Memoriam: Jerry B. Hook (1938 –2021)

**DOI:** 10.1093/toxsci/kfab014

**Published:** 2021-02-14

**Authors:** Jay I Goodman, Norbert E Kaminski

**Affiliations:** 1 Department of Pharmacology & Toxicology, Michigan State University, East Lansing, Michigan 48824, USA; 2 Department of Pharmacology & Toxicology, Institute for Integrative Toxicology, Center for Research on Ingredient Safety, Michigan State University, East Lansing, Michigan 48824, USA


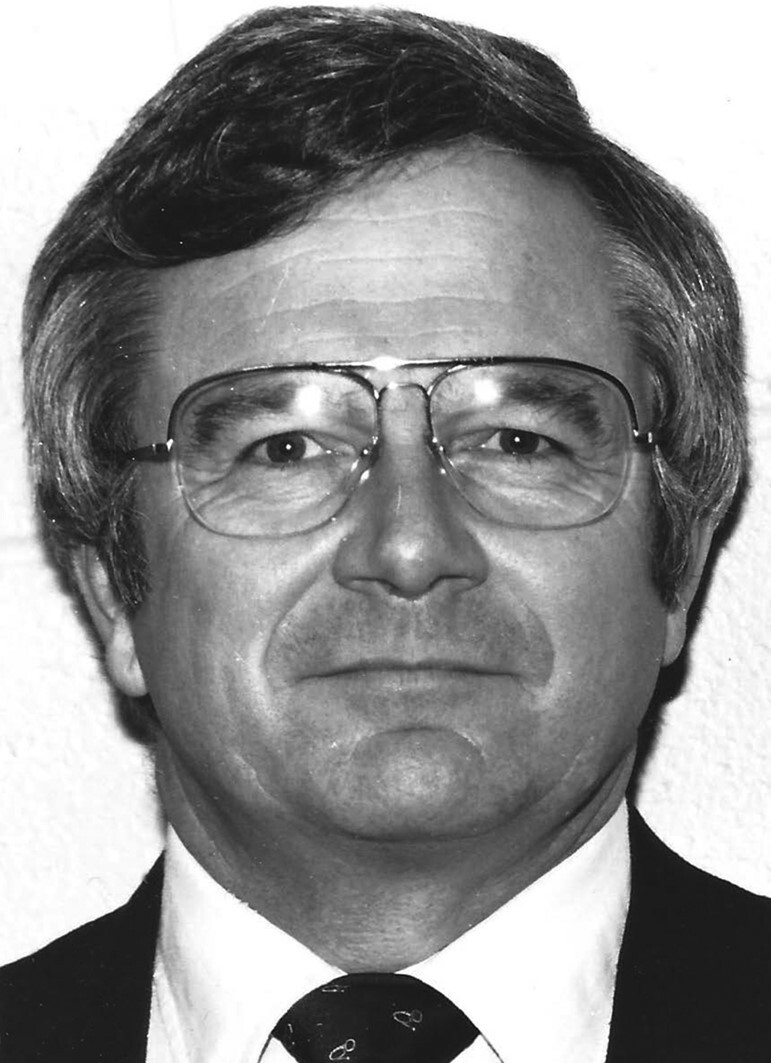


Jerry was a very well-known and highly respected Pharmacologist and Toxicologist whose research spanned the areas of mechanisms of drug action on the kidney, to the ontogeny of renal transport systems, to mechanisms of renal toxicity. He was critical in advancing the field of toxicology by helping to drive it from observational to a mechanism-based science. Jerry published more than 250 papers, review articles, and book chapters. He was the Editor of Toxicology of the Kidney (Target Organ Toxicology Series), 1981, and in collaboration with Robin Goldstein (who performed her doctoral thesis research in Jerry’s laboratory), co-edited the second edition of the book, 1992.

Jerry received his PhD in Pharmacology from the University of Iowa in 1966 and later that year was recruited to join the faculty at Michigan State University (MSU) as an Assistant Professor in the new Department of Pharmacology. He contributed substantially to the department’s toxicology expertise that led to its evolving to the Department of Pharmacology and Toxicology in 1978. He was an extensive collaborator and worked effectively across disciplines and departments. Jerry was named Founding Director of MSU’s Center for Environmental Toxicology (CET) in 1981 and successfully developed multidisciplinary approaches aimed at resolving human and environmental toxicology issues. Jerry’s achievements were the foundation for the CET to develop into today’s Institute for Integrative Toxicology. Jerry served on the Editorial Boards of numerous journals and was a member of a number of advisory committees, notably the National Toxicology Program’s Board of Scientific Councilors where he Chaired its Peer Review Panel. He was very active in a variety of scientific societies, including the American Society for Pharmacology and Experimental Therapeutics, the International Union of Pharmacology, the International Union of Toxicology, and the Society of Toxicology, where he was President from 1987 to 1988.

Jerry was regarded highly as a mentor and had a profound influence on graduate education in toxicology at MSU. Over the course of his 17 years as a faculty member, 5 post-doctoral fellows, 10 PhD students, and 4 MS students received their training in his laboratory. The Department’s faculty considered him a caring and supportive colleague, always eager to engage in scientific discussion and share his knowledge/insight, with a particular passion for encouraging trainees. We were always proud to say, “Jerry Hook is my friend.” Indeed, Jerry is regarded as an icon of MSU’s Department of Pharmacology & Toxicology and of the field of toxicology.

In 1983, Jerry was recruited to join Smith Kline & French Laboratories (later becoming Smith Kline Beecham) as Vice President for Preclinical Research and Development, where he directed laboratories in the United States and United Kingdom, and rose to Senior Vice President and Director of Development. In 1993, he founded Lexin Pharmaceutical Company (LPC), a biotechnology company, and served as its CEO. Three years later, LPC merged into Sparta Pharmaceuticals, Inc. which Jerry headed until his retirement in 1999.

While we mourn the loss of Jerry, it is important to remember that the discipline of Toxicology has been enhanced by his pioneering contributions to our understanding of mechanisms underlying chemical-induced renal toxicity. We who worked with, or knew, Jerry are richer for having known the man, and it is our hope that he knew how much he was appreciated.

## Notes

**In Memoriam: Jerry B. Hook** (1938–2021).

Dr. Jerry B. Hook passed on January 9, 2021. We express our heartfelt condolences to his wife, Dr. Jacqueline H. Smith, and all of the Hook family.

